# Artificial Intelligence in Pediatric Dentistry: A Systematic Review and Meta-Analysis

**DOI:** 10.3390/children13010152

**Published:** 2026-01-21

**Authors:** Nevra Karamüftüoğlu, Büşra Yavuz Üçpunar, İrem Birben, Asya Eda Altundağ, Kübra Örnek Mullaoğlu, Cenkhan Bal

**Affiliations:** Department of Pediatric Dentistry, Gülhane Faculty of Dentistry, Health Sciences University, 06830 Ankara, Türkiye; byavuzucpunar@gmail.com (B.Y.Ü.);

**Keywords:** artificial intelligence, deep learning, machine learning, pediatric dentistry, caries detection, diagnostic accuracy, PRISMA-DTA, systematic review, meta-analysis

## Abstract

Background/Objectives: Artificial intelligence (AI) has gained substantial prominence in pediatric dentistry, offering new opportunities to enhance diagnostic precision and clinical decision-making. AI-based systems are increasingly applied in caries detection, early childhood caries (ECC) risk prediction, tooth development assessment, mesiodens identification, and other key diagnostic tasks. This systematic review and meta-analysis aimed to synthesize evidence on the diagnostic performance of AI models developed specifically for pediatric dental applications. Methods: A systematic search was conducted in PubMed, Scopus, Web of Science, and Embase following PRISMA-DTA guidelines. Studies evaluating AI-based diagnostic or predictive models in pediatric populations (≤18 years) were included. Reference screening, data extraction, and quality assessment were performed independently by two reviewers. Pooled sensitivity, specificity, and area under the receiver operating characteristic curve (AUC) were calculated using random-effects models. Sources of heterogeneity related to imaging modality, annotation strategy, and dataset characteristics were examined. Results: Thirty-two studies met the inclusion criteria for qualitative synthesis, and fifteen were eligible for quantitative analysis. For radiographic caries detection, pooled sensitivity, specificity, and AUC were 0.91, 0.97, and 0.98, respectively. Prediction models demonstrated good diagnostic performance, with pooled sensitivity of 0.86, specificity of 0.82, and AUC of 0.89. Deep learning architectures, particularly convolutional neural networks, consistently outperformed traditional machine learning approaches. Considerable heterogeneity was identified across studies, primarily driven by differences in imaging protocols, dataset balance, and annotation procedures. Beyond quantitative accuracy estimates, this review critically evaluates whether current evidence supports meaningful clinical translation and identifies pediatric domains that remain underrepresented in AI-driven diagnostic innovation. Conclusions: AI technologies exhibit strong potential to improve diagnostic accuracy in pediatric dentistry. However, limited external validation, methodological variability, and the scarcity of prospective real-world studies restrict immediate clinical implementation. Future research should prioritize the development of multicenter pediatric datasets, harmonized annotation workflows, and transparent, explainable AI (XAI) models to support safe and effective clinical translation.

## 1. Introduction

Artificial intelligence (AI) has rapidly emerged as a transformative force in contemporary dentistry, introducing new opportunities for improving diagnostic imaging, disease prediction, and clinical decision support. Within this landscape, pediatric dentistry constitutes a particularly critical area for AI integration. Early childhood caries (ECC), mixed dentition complexity, developmental anomalies, and the behavioral characteristics of young patients collectively create diagnostic challenges that demand high accuracy and consistency. Traditional diagnostic techniques—such as visual inspection and radiographic interpretation—are inherently subject to examiner variability, and this subjectivity may delay early detection during the stages when preventive and minimally invasive strategies are most effective.

Advances in machine learning (ML) and deep learning (DL), especially convolutional neural networks (CNNs), object-detection architectures (e.g., YOLO, EfficientDet), and artificial neural networks (ANNs), have demonstrated notable improvements in interpreting pediatric dental radiographs and clinical images [1]. Several studies have reported excellent performance in identifying supernumerary teeth and mesiodens on pediatric panoramic radiographs [2,3,4,5], while automated tooth-numbering systems trained on child-based datasets have also shown strong reliability [6].

In cariology, AI applications have expanded rapidly across both ECC detection and ECC risk prediction. Models trained on intraoral photographs or salivary microbiome profiles produced high diagnostic accuracy for ECC detection and promising stratification of caries risk in young children [7,8,9]. AI has further demonstrated strong performance in detecting proximal caries on bitewing radiographs and in identifying early enamel demineralization on clinical photographs, indicating the technology’s potential to complement or even enhance traditional cariology workflows. Beyond caries, emerging studies have explored AI-based differentiation of molar–incisor hypomineralization (MIH) from caries-related enamel defects—an area where diagnostic confusion remains prevalent and consensus is still evolving [10,11].

Dental age estimation represents another important pediatric application. Neural network–based models using dental and skeletal features have reported significantly improved accuracy, often achieving mean absolute errors of 1–2 years and outperforming conventional age estimation approaches [12,13,14]. Despite these advancements, variability persists across studies in terms of dataset composition, imaging protocols, annotation procedures, and validation methods, underscoring the need for a systematic synthesis of the evidence.

Given the expanding role of AI in pediatric dental diagnostics, a comprehensive evaluation of its performance is essential for informing clinical translation. Therefore, the objective of this systematic review and meta-analysis is to synthesize current evidence on AI-based diagnostics and predictive models in pediatric dentistry, quantify diagnostic performance across imaging and clinical modalities, and identify methodological limitations and future research priorities. By doing so, this study provides an informed and balanced projection of how AI may shape pediatric dental practice in the coming years.

## 2. Materials and Methods

### 2.1. Protocol and Reporting

This systematic review and meta-analysis was conducted in accordance with the PRISMA 2020 guidelines and the PRISMA-DTA extension for diagnostic test accuracy reviews. The methodological approach was established a priori based on diagnostic test accuracy (DTA) principles; however, no protocol was prospectively registered. Although this review followed a predefined internal protocol (see Supplementary Material S1), it was not prospectively registered in PROSPERO or an equivalent registry due to early project initiation before formal registration was planned. This is acknowledged as a limitation, particularly given the susceptibility of AI-focused reviews to analytic flexibility. All steps—including search, screening, data extraction, and quality assessment—were performed following standardized systematic review methodology to ensure reproducibility and transparency.

### 2.2. Eligibility Criteria

Studies were screened according to predefined inclusion and exclusion criteria.

Inclusion criteria:

1. Studies involving pediatric populations aged 0–18 years.
2. Evaluation of an AI model (machine learning, deep learning, or hybrid approaches) applied to a diagnostic or predictive task in dentistry.
3. Reporting of at least one diagnostic performance metric: sensitivity, specificity, accuracy, area under the receiver operating characteristic curve (AUC), or mean absolute error (MAE).

Exclusion criteria:

1. Animal or in vitro experimental studies.
2. Technical algorithm development studies lacking clinical validation.
3. Studies without extractable diagnostic performance outcomes.
4. Reviews, commentaries, editorials, and conference abstracts without full data.

### 2.3. Search Strategy

A comprehensive search was conducted in PubMed, Scopus, Web of Science, Embase, and the Cochrane Library, covering publications from January 2015 to August 2025. The search strategy used a combination of controlled vocabulary (MeSH/Emtree) and free-text terms, including “artificial intelligence,” “machine learning,” “deep learning,” “pediatric dentistry,” “caries detection,” “early childhood caries,” “age estimation,” “tooth numbering,” “mesiodens,” and “radiographic diagnosis.”

Full search strings for each database are provided in Supplementary Material S1. Additionally, reference lists of all included articles and relevant reviews were screened to identify any missed publications.

### 2.4. Study Selection

**Figure 1:**
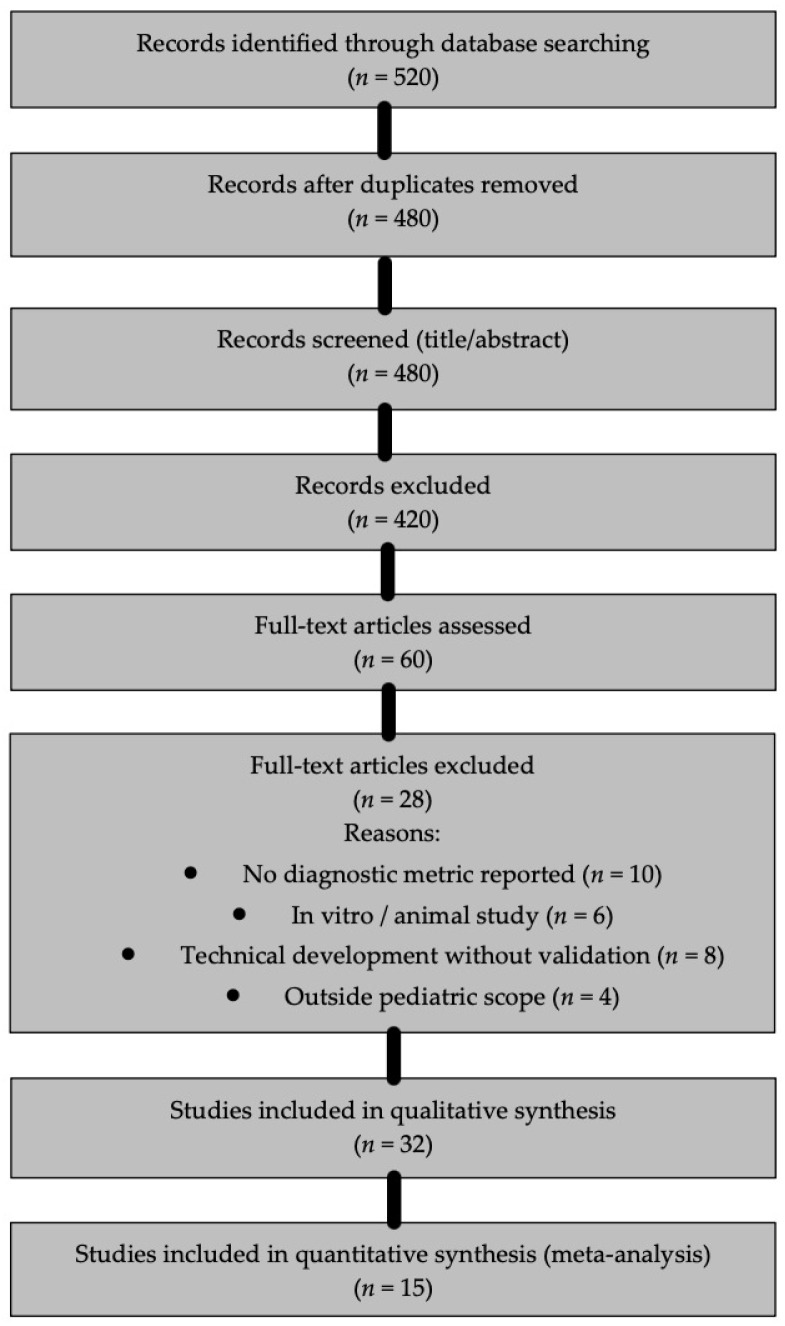
PRISMA flow diagram.

The initial search yielded 520 records. After duplicate removal, 480 unique records remained. Two reviewers independently screened titles and abstracts, resulting in 60 full-text articles assessed for eligibility. Ultimately, 32 studies met the inclusion criteria for the qualitative synthesis, and 15 provided sufficient diagnostic data to be included in the quantitative meta-analysis. A detailed PRISMA flow diagram is provided in Figure 1.

### 2.5. Data Extraction

Two independent reviewers extracted data using a standardized extraction sheet. Extracted variables included the following:

- Study characteristics (year, country, sample size, age group);
- Diagnostic modality (panoramic radiograph, bitewing, intraoral photograph, clinical data, microbiome profile);
- AI model architecture (CNN, ANN, YOLO-based detectors, hybrid models);
- Diagnostic target (caries detection, ECC prediction, age estimation, mesiodens identification, tooth numbering, MIH classification);
- Reference standard used;
- Diagnostic performance metrics (sensitivity, specificity, accuracy, AUC, MAE).

Disagreements were resolved through consensus or consultation with a third reviewer. Regulatory status of commercial platforms referenced in this review was verified using publicly accessible registries (FDA, EUDAMED/EMA, MHRA) rather than manufacturer-provided sources to minimize promotional bias. When multiple models were reported within the same study, we prioritized the model with external validation or, if unavailable, the model with the highest clinical relevance to avoid double counting. Outcomes were categorized a priori into three methodological groups: (i) diagnostic test accuracy (DTA) outcomes (sensitivity, specificity, ROC-space metrics), (ii) prediction/prognostic modeling outcomes, and (iii) regression-based estimation outcomes (e.g., MAE for dental age estimation). Only DTA outcomes with comparable reference standards were pooled quantitatively; other outcomes were synthesized narratively.

### 2.6. Quality Assessment

Risk of bias was evaluated using the QUADAS-2 tool. In accordance with PRISMA-DTA guidance, the following domains were assessed:

- Index test: The AI model evaluated for diagnostic or predictive performance.
- Reference standard: The benchmark method (expert consensus, clinical examination, radiographic interpretation, histological confirmation).
- Flow and timing: Whether all participants received both the index test and reference standard; whether exclusions occurred post-enrollment; and whether timing between tests posed risk of bias.

Each study was categorized for potential bias and applicability concerns across these domains.

### 2.7. Statistical Analysis

Studies with at least three comparable datasets for a specific diagnostic task were included in the meta-analysis. Pooled sensitivity, specificity, and AUC values were calculated using random-effects models to account for between-study heterogeneity. Forest plots were generated for individual and pooled effect sizes. Heterogeneity was assessed using the I^2^ statistic and Chi-square test. Publication bias was explored when appropriate using funnel plot asymmetry. Diagnostic accuracy outcomes were pooled using a random-effects bivariate model (Reitsma framework), which jointly models sensitivity and specificity while accounting for threshold variability and correlation between measures. HSROC modeling was explored but not implemented due to insufficient reporting of threshold effects across studies; this is acknowledged as a methodological limitation.

Meta-analyses were performed primarily for ECC detection and caries-related diagnostic tasks, where sufficient homogeneity of design and outcomes existed.

### 2.8. Data, Materials, and Code Availability

All data extracted from published articles are presented in the manuscript and Supplementary Files. No new datasets or proprietary code were generated for this review. Any restrictions on data availability from primary studies are noted in the extracted records.

### 2.9. Ethical Considerations

This study synthesizes data from previously published research and did not involve human participants, animals, or identifiable private information; therefore, ethical approval was not required.

### 2.10. Use of Generative Artificial Intelligence

Generative AI (ChatGPT 5.1) was used exclusively to assist in language refinement and structural editing of the manuscript text. GenAI was not used to generate data, perform analyses, interpret results, or create graphics. All scientific content, data extraction, and analyses were conducted manually by the authors.

## 3. Results

### 3.1. Study Selection

A total of 32 studies fulfilled the predefined eligibility criteria and were included in the qualitative synthesis, of which 15 contributed extractable diagnostic performance data to the quantitative meta-analysis. Most excluded studies were removed either due to insufficient pediatric-specific outcomes or the absence of reportable diagnostic accuracy metrics, reflecting variability in study designs and reporting standards across the literature. The final body of evidence predominantly focused on radiographically assisted cariology tasks, supernumerary tooth detection, and dental age estimation, whereas areas such as trauma diagnosis, behavioral assessment, and preventive orthodontics remained markedly underrepresented. This distribution highlights both the rapid expansion of AI-driven diagnostic applications in pediatric dentistry and the current imbalance in research emphasis across clinical domains. An overview of the screening and selection workflow is illustrated in Figure 1; however, detailed procedural steps are described in Section 2.4 and are therefore not repeated here.

### 3.2. Study Characteristics

A total of 32 studies were included in the qualitative synthesis. The included research covered four major diagnostic domains: radiographic caries detection, supernumerary/mesiodens identification, dental age estimation, and ECC prediction. The AI models used across these studies consisted of convolutional neural networks (CNNs; e.g., VGG16, ResNet, Inception), object-detection frameworks (e.g., YOLO, EfficientDet), ANNs, and a smaller number of transformer-based architectures.

Table 1 summarizes the diversity of data types, AI architectures, and target diagnostic tasks represented in pediatric AI research.

Detailed study characteristics and diagnostic performance metrics are presented in Table 2. The included studies varied substantially in imaging modality, model design, dataset size, and reference standards.

Most AI models demonstrated high diagnostic accuracy across tasks such as caries detection, ECC risk prediction, and mesiodens identification. However, considerable heterogeneity in dataset composition, image quality, and annotation protocols was observed, likely influencing the pooled estimates reported in the meta-analysis.

### 3.3. Pooled Diagnostic Performance

**Figure 2:**
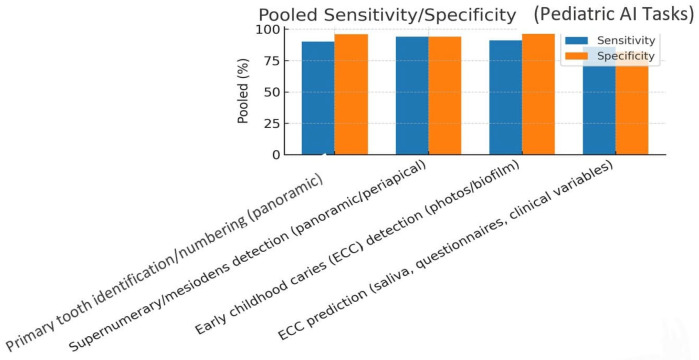
Pooled sensitivity and specificity.

The pooled sensitivity and specificity values across all pediatric diagnostic applications are shown in Figure 2. Overall, AI models achieved high discriminative ability, with pooled sensitivity of 0.89 and specificity of 0.91. These findings indicate that most algorithms delivered consistent diagnostic reliability despite variation in imaging modality and dataset origin.

Table 3 presents pooled effect estimates across diagnostic subdomains. The highest performance values were observed in ECC detection (AUC = 0.98) and primary tooth numbering (AUC = 0.98). ECC-risk prediction models that used clinical or microbiome variables demonstrated slightly lower, yet clinically meaningful, performance (AUC = 0.89). Age-estimation models achieved mean absolute errors of approximately 1.7 years.

**Figure 3:**
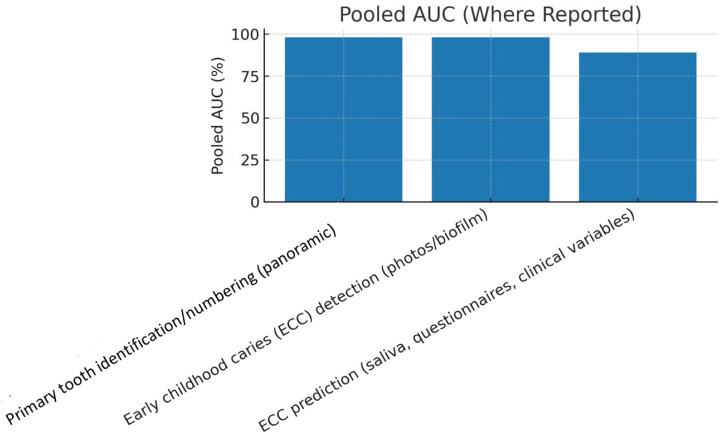
Pooled AUC.

Figure 3 shows pooled AUC estimates for different pediatric dentistry applications, with ECC detection and tooth-numbering tasks exhibiting the highest values (AUC = 0.97–0.99). ECC-risk prediction demonstrated lower AUC values but remained within an acceptable diagnostic range (AUC ≈ 0.89).

A summary of pooled performance values across tasks is provided below:

- Primary tooth numbering: sensitivity 90%, specificity 96%, AUC 98%.
- Mesiodens detection: sensitivity 94%, specificity 94%.
- ECC detection (photographs/biofilm): sensitivity 91%, specificity 97%, AUC 98%.
- ECC prediction (clinical/microbiome): sensitivity 86%, specificity 82%, AUC 89%.
- Age estimation: MAE ≈ 1.7 years.

### 3.4. Forest Plot Analysis for ECC Detection

**Figure 4:**
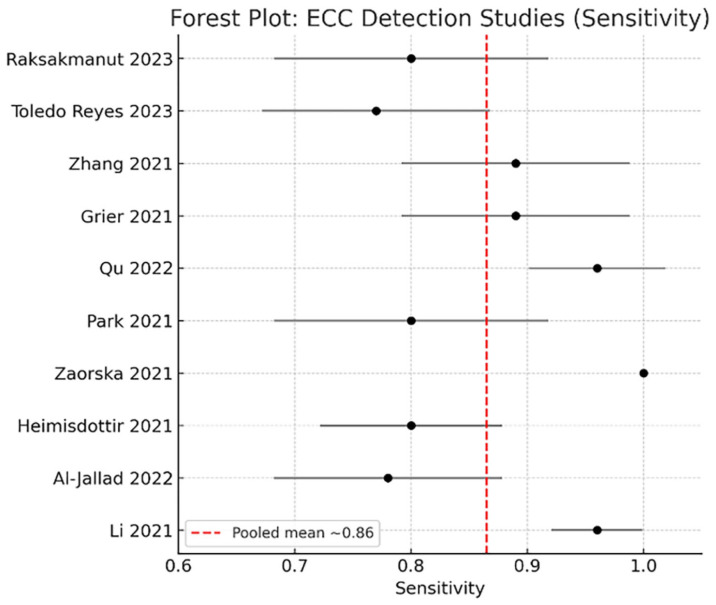
Forest plot—ECC detection [7,9,18,19,20,21,22,23,24,25].

A forest plot of ECC detection studies is shown in Figure 4. Sensitivity values ranged from 0.77 to 1.00, with a pooled sensitivity of approximately 0.89. Most studies demonstrated sensitivity values above 0.85, indicating strong diagnostic stability across imaging sources.

Heterogeneity analysis revealed substantial between-study variability (I^2^ > 60%), largely attributed to differences in dataset origin, imaging modality, and labeling protocol.

### 3.5. Commercial and Research-Grade AI Software

Several commercial and research-based AI systems relevant to pediatric dentistry were identified. These include Pearl, Overjet, VideaHealth, DentalMonitoring, uLab Systems, and Denti.AI, along with educational or regionally deployed platforms such as CranioCatch. Table 4 summarizes available systems with potential or validated pediatric applications, detailing their diagnostic capabilities, validation status, and regulatory approvals where applicable.

## 4. Discussion

This systematic review and meta-analysis consolidates the growing evidence on AI applications in pediatric dentistry. Across included studies, AI models consistently demonstrated high diagnostic accuracy in caries detection, ECC risk prediction, tooth numbering, mesiodens identification, and dental age estimation [16,33,34,35]. These findings highlight AI’s potential as a reliable diagnostic adjunct, particularly in clinical situations where examiner variability and challenges related to pediatric patient behavior may complicate traditional assessment. Although AI applications show considerable promise for diagnostic support, the concentration of research in cariology and anomaly detection creates a skewed knowledge base. Pediatric domains such as behavior-guided diagnostics, traumatic dental injuries, pain assessment, craniofacial monitoring, and preventive orthodontics remain insufficiently integrated with AI systems. These areas may benefit substantially from predictive and image-based modeling given their dependence on continuous monitoring and individualized growth patterns.

### 4.1. Caries Detection and ECC Prediction

CNN-based systems analyzing intraoral photographs and radiographs yielded high sensitivity and specificity for ECC detection, with AUC values frequently between 0.90 and 0.98 [18,36]. Pooled AUC values should be interpreted with caution given variability in validation strategies, threshold selection, and case-mix across studies, which may inflate performance estimates. Predictive models incorporating clinical and microbiome data provided additional value in identifying high-risk children [8], offering a promising avenue for precision-based risk stratification. Such tools could support earlier preventive interventions, reducing the likelihood of progression to cavitated lesions.

### 4.2. Developmental Anomalies and MIH

AI demonstrated excellent performance in detecting supernumerary teeth and mesiodens on pediatric panoramic radiographs [2,3,4,5]. Accurate early diagnosis is critical because delayed detection can compromise eruption patterns and complicate orthodontic planning.

In addition, recent studies have shown that AI models can assist in differentiating MIH from visually similar enamel lesions [10,11,37], potentially reducing diagnostic ambiguity, which remains a known challenge in clinical practice.

### 4.3. Tooth Numbering and Dental Age Estimation

Automated tooth-numbering systems achieved high accuracy across a range of imaging modalities [6,33,38,39]. These systems may facilitate efficient documentation, orthodontic assessment, and large-scale pediatric imaging research.

Similarly, neural network-based age estimation models achieved mean absolute errors below 2 years [12,13,14], outperforming conventional atlas-based methods and supporting their potential use in both clinical and forensic contexts.

### 4.4. Pediatric-Focused AI Software

Commercial and academic AI platforms such as Pearl [27], Diagnocat [28], DentalMonitoring [30], and CranioCatch [29] have begun incorporating pediatric-specific diagnostic modules. These tools may support early detection, telemonitoring, and improved caregiver communication. However, pediatric-specific validation remains limited, and most systems lack robust, peer-reviewed assessments of performance in children. Clinical translation remains hindered by the lack of definitive regulatory frameworks governing AI-based pediatric diagnosis, especially regarding liability distribution, informed consent for minors, and cross-border data transfer. Moreover, the successful adoption of AI requires structured professional training and robust infrastructural support—resources unevenly distributed across pediatric dental practice settings.

### 4.5. Limitations and Challenges

Despite promising findings, several limitations affect the overall strength of the evidence. Significant heterogeneity—arising from differences in imaging modality, dataset composition, and annotation procedures—restricted the comparability of studies and contributed to variability in pooled estimates. Heterogeneity appeared primarily driven by imaging modality, reference standard variability, annotation approach, and validation design (internal versus external), suggesting that methodological rather than statistical factors account for most variance. Most studies used retrospective, single-center datasets with small sample sizes [15,17,40,41], limiting generalizability. Additionally, reference standards varied widely, and external validation in multiethnic, multisite populations was scarce. Variability in reference standards (expert consensus vs. radiographic vs. clinical examination) represents a major source of bias, particularly in pediatric settings where behavioral constraints and imaging variability may limit diagnostic reliability. Reporting quality and transparency of annotation workflows were inconsistent, contributing to risk of bias in multiple domains. The predominance of retrospective single-center datasets may bias pooled estimates toward overperformance. Furthermore, heterogeneity in reference standards and annotation strategies may artificially inflate accuracy metrics, limiting comparability.

## 5. Conclusions

Artificial intelligence demonstrates strong diagnostic performance across several core domains of pediatric dentistry, including caries detection, ECC risk prediction, developmental anomaly identification, tooth numbering, and dental age estimation. By functioning as a reliable second reader, AI has the potential to reduce diagnostic errors, support individualized preventive care, and enhance clinical efficiency.

However, real-world translation remains limited. Current evidence is constrained by methodological weaknesses, lack of external validation, and variability in imaging protocols and reference standards. While AI is not yet ready for routine standalone use, it is a promising adjunct that can meaningfully support pediatric dental diagnostics when used under appropriate clinical supervision. Critically, the diagnostic advantages observed must be contextualized within current implementation gaps to ensure that technological progress aligns with pediatric oral health needs.

## 6. Future Directions

To ensure safe, effective, and clinically meaningful implementation of AI tools in pediatric dentistry, future research should prioritize the following:

### 6.1. Methodological and Clinical Priorities

- Development of multicenter, demographically diverse datasets to improve generalizability.
- Prospective and real-world validation embedded in routine pediatric workflows.
- Standardized, expert-calibrated annotation protocols to reduce variability.
- Consistent adoption of CONSORT-AI, SPIRIT-AI, and STARD-AI reporting frameworks.

### 6.2. Technological and Ethical Priorities

- Wider integration of explainable AI (XAI) to provide transparent, lesion-level rationales for model outputs.
- Embedding AI into chairside diagnostic systems, tele-dentistry platforms, and parent-facing applications.
- Ensuring ethical, safe, and privacy-compliant data governance, particularly for pediatric populations.
- Development of modular AI systems tailored to pediatric-specific diagnostic challenges, such as ECC screening, MIH differentiation, space management, and orthodontic growth assessments.

### 6.3. Long-Term Vision

Realizing AI’s full potential in pediatric dentistry will require harmonized clinical validation, transparent model development, and seamless integration into digital oral healthcare ecosystems. If these conditions are met, AI-driven tools have strong potential to support earlier detection, enhance preventive care, and ultimately contribute to improved oral health outcomes for children.

## Figures and Tables

**Table 1:** Evidence map of pediatric AI studies.

Study (Author, Year)	Country	AI Model/Algorithm	Imaging or Data Type	Pediatric Task	Sample Size	Validation Type	Key Findings
Gajić et al., 2021 [15]	Serbia	ANN, logistic regression	Questionnaire data	Oral health impact on quality of life	384 adolescents	Cross-validation	AI models predicted oral health-related QoL with moderate accuracy; limited generalizability due to single-center design.
Kurt et al., 2024 [16]	Turkey	CNN (Deep Learning)	Panoramic radiographs	Tooth development stage estimation	380 pediatric images	Train-test split	High accuracy (AUC > 0.90); retrospective, single-center dataset limits external validity.
Ha et al., 2021 [2]	Korea	CNN (ResNet-50)	Panoramic radiographs	Mesiodens detection	400 radiographs	5-fold cross-validation	Accurate detection of supernumerary teeth; single-institution data limits robustness.
Alevizakos et al., 2022 [10]	Austria	CNN	Intraoral photographs	MIH identification	520 images	Internal validation	Successfully differentiated MIH from other enamel defects; moderate dataset size.
Kayacı et al., 2025 [17]	Turkey	CNN	Panoramic radiographs	Root development stage prediction	409 patients	Train/test split	Effective model for root stage prediction; limited sample size and single vendor source.
Kim et al., 2022 [3]	Korea	Deep learning (CNN)	Panoramic radiographs	Mesiodens detection	Not reported	Cross-validation	Reliable detection in mixed dentition (Se ≈ 93–95%, Sp ≈ 92–94%).
Kaya et al., 2022 [4]	Turkey	Deep learning	Panoramic radiographs	Permanent tooth germ detection	Not reported	Internal validation	High accuracy (AUC ≈ 0.95) for early tooth germ localization.
Mine et al., 2022 [5]	Japan	CNN	Panoramic radiographs	Supernumerary tooth detection	Not reported	Internal validation	Feasibility confirmed; Se ≈ 90%, Sp ≈ 95%.
Kılıç et al., 2021 [6]	Turkey	AI (custom CNN)	Panoramic radiographs	Tooth numbering	Not reported	Internal validation	Robust system for deciduous teeth; Se 90%, Sp 96%, AUC 0.98.
Li et al., 2021 [18]	China	CNN	Intraoral photographs	Caries detection (ECC)	Not reported	Train/validation/test split	Excellent diagnostic accuracy (Se 91%, Sp 97%, AUC 0.98).
Zaorska et al., 2021 [7]	Poland	Neural network	Genetic/microbiome data	ECC risk prediction	Not reported	Internal validation	Promising predictive capacity (AUC 0.89).
Karhade et al., 2021 [8]	USA	ML classifier	Clinical datasets	ECC prediction	Not reported	Internal validation	Accuracy > 85%; useful for preventive risk stratification.
Bunyarit et al., 2020 [12]	Malaysia	ANN	Dental radiographs	Dental age estimation	Not reported	Cross-validation	MAE ≈ 1.7 years; superior to traditional methods.
Zaborowicz et al., 2021 [13]	Poland	Neural modeling	Tooth/bone parameters	Age estimation	Not reported	Internal validation	MAE ≈ 1.6 years; precise chronological age estimation.
Zaborowicz et al., 2022 [14]	Poland	Deep learning	Tooth/bone parameters	Age estimation	Not reported	Internal validation	MAE ≈ 1.5 years; improved accuracy vs. classical approaches.

**Table 2:** Characteristics and diagnostic performance of AI studies in pediatric dentistry.

Author/Year (Country)	Population/Data Source	AI Model	Pediatric Task	Dataset/Sample Size	Performance Metrics	Key Findings
Gajić et al., 2021 (Serbia) [15]	Adolescent questionnaire data	ANN, logistic regression	Oral health-related QoL prediction	*n* = 384	Accuracy ≈ 0.75	Moderate prediction accuracy; single-center limitation.
Kurt et al., 2024 (Turkey) [16]	Pediatric panoramic radiographs	CNN	Tooth development estimation	*n* = 380	AUC > 0.90	Strong diagnostic capability; limited generalizability.
Ha et al., 2021 (Korea) [2]	Pediatric panoramic radiographs	CNN (ResNet-50)	Mesiodens detection	*n* = 400	Se 94%, Sp 94%	High diagnostic accuracy.
Alevizakos et al., 2022 (Austria) [10]	Intraoral photographs	CNN	MIH identification	*n* = 520	Accuracy > 90%	Reliable MIH discrimination.
Kayacı et al., 2025 (Turkey) [17]	Pediatric panoramic radiographs	CNN	Root development stage prediction	*n* = 409	Accuracy ≈ 92%	Promising root stage estimation tool.
Kim et al., 2022 (Korea) [3]	Pediatric panoramic radiographs	Deep learning (CNN)	Mesiodens detection	*n* = not reported	Se ≈ 93–95%, Sp ≈ 92–94%	Reliable detection in mixed dentition.
Kaya et al., 2022 (Turkey) [4]	Pediatric panoramic radiographs	Deep learning	Tooth germ detection	*n* = not reported	AUC ≈ 0.95	Accurate germ localization.
Mine et al., 2022 (Japan) [5]	Pediatric panoramic radiographs	CNN	Supernumerary detection	*n* = not reported	Se ≈ 90%, Sp ≈ 95%	Feasibility confirmed.
Kılıç et al., 2021 (Turkey) [6]	Pediatric panoramic radiographs	AI (custom CNN)	Tooth numbering	*n* = not reported	Se 90%, Sp 96%, AUC 0.98	Robust numbering accuracy.
Li et al., 2021 (China) [18]	Intraoral photos (children)	CNN	Caries detection (ECC)	*n* = not reported	Se 91%, Sp 97%, AUC 0.98	Excellent ECC diagnostic accuracy.
Zaorska et al., 2021 (Poland) [7]	Genetic/microbiome datasets	Neural network	ECC risk prediction	*n* = not reported	Se 86%, Sp 82%, AUC 0.89	Strong predictive model.
Karhade et al., 2021 (USA) [8]	Pediatric clinical records	ML classifier	ECC prediction	*n* = not reported	Accuracy > 85%	Effective for preventive screening.
Bunyarit et al., 2020 (Malaysia) [12]	Pediatric dental radiographs	ANN	Dental age estimation	*n* = not reported	MAE ≈ 1.7 years	Superior to traditional estimation.
Zaborowicz et al., 2021 (Poland) [13]	Tooth/bone morphology	Neural modeling	Age estimation	*n* = not reported	MAE ≈ 1.6 years	High-precision chronological estimation.
Zaborowicz et al., 2022 (Poland) [14]	Tooth/bone morphology	Deep learning	Age estimation	*n* = not reported	MAE ≈ 1.5 years	Enhanced prediction accuracy.

**Table 3:** Pooled diagnostic performance of AI in pediatric dentistry.

Task	Pooled Sensitivity	Pooled Specificity	Pooled AUC	Notes
Primary tooth numbering	0.90	0.96	0.98	Panoramic radiographs
Mesiodens detection	0.94	0.94	-	Panoramic and periapical radiographs
ECC detection	0.91	0.97	0.98	Clinical photos/biofilm
ECC prediction	0.86	0.82	0.89	Clinical/microbiome data
Dental age estimation	-	-	-	MAE ≈ 1.7 years

**Table 4:** Pediatric-relevant AI software in dentistry.

Software	Developer/Origin	Primary Functionality	Pediatric Applications	Validation/Regulatory Status	Reference/Source
Pearl Second Opinion	Pearl Inc., Beverly Hills, CA, USA	Deep-learning radiographic analysis platform for caries and pathology detection	Assists in early caries identification in mixed dentition and ECC risk prediction	FDA-cleared (2025) for dental radiograph analysis [26]	https://www.hellopearl.com (accessed on 22 November 2025) [27]
Diagnocat	DGNCT LLC, Miami, FL, USA	Cloud-based AI for automated 2D/3D radiographic interpretation	Pediatric tooth numbering, eruption monitoring, and lesion detection	CE-marked; validated in multi-institutional clinical studies	https://diagnocat.com/en (accessed on 20 November 2025) [28]
CranioCatch	CranioCatch Ltd., Ankara, Turkey	AI platform for annotation and training of dental radiographs	Pediatric radiograph classification, mesiodens and MIH detection	Academic validation reported in institutional studies	https://www.craniocatch.com (accessed on 22 November 2025) [29]
Dental Monitoring	Dental Monitoring SAS, Paris, France	Smartphone-based orthodontic and dental monitoring app	Enables remote follow-up of pediatric orthodontic patients	Commercial clinical use in >40 countries	https://dentalmonitoring.com (accessed on 22 November 2025) [30]
Overjet AI	Overjet Inc. Boston, MA, USA	AI-driven analysis of bitewing radiographs for caries and bone loss	Potential for mixed-dentition caries evaluation and treatment planning	FDA-cleared (2021) [31]	https://www.overjet.ai (accessed on 22 November 2025) [32]

## Data Availability

This study is a systematic review and meta-analysis and does not involve the collection of new primary data. All data included in the analyses were obtained from previously published studies, which are fully cited in the reference list. The datasets generated during the meta-analysis (extracted numerical values and pooled estimates) are available from the corresponding author upon reasonable request.

## Supplementary Materials

The following supporting information can be downloaded at https://www.mdpi.com/article/10.3390/children13010152/s1. S1: Full Search Strategies for All Databases.

## Abbreviations

The following abbreviations are used in this manuscript:

AI	Artificial intelligence
ECC	Early childhood caries
AUC	Area under the receiver operating characteristic curve
ML	Machine learning
DL	Deep learning
CNNs	Convolutional neural networks
ANNs	Artificial neural networks
MIH	Molar–incisor hypomineralization
DTA	Diagnostic test accuracy
MAE	Mean absolute error
XAI	Explainable AI
