# Supplementary material for: Artificial Intelligence in Pediatric Dentistry: A Systematic Review and Meta-Analysis
Source: Children (Basel). 2026 Jan 21;13(1):152. doi: 10.3390/children13010152 (PMC12839933; doi:10.3390/children13010152)
Supplement: Supplementary file 1 [file children-13-00152-s001.zip › children-4032870-supplementary.pdf]

Supplementary Material S1: Full Search Strategies for All Databases

This supplementary file provides the complete search strategies used for each database included in this systematic review and meta-analysis. Searches covered publications from January 2015 to August 2025. Both controlled vocabulary (MeSH/Emtree) and free-text terms were used.

1. PubMed (MEDLINE) Search Strategy

(("artificial intelligence"[MeSH Terms] OR "machine learning"[MeSH Terms]
OR "deep learning"[MeSH Terms] OR "neural networks (computer)"[MeSH Terms]
OR "artificial intelligence"[tiab] OR "machine learning"[tiab]
OR "deep learning"[tiab] OR "neural network*"[tiab] OR AI[tiab])
AND
("pediatric dentistry"[MeSH Terms] OR "child"[MeSH Terms]
OR children[tiab] OR pediatric[tiab] OR paediatric[tiab]
OR "pediatric dentistry"[tiab] OR "paediatric dentistry"[tiab])
AND
(caries[MeSH Terms] OR "dental caries"[tiab] OR "early childhood caries"[tiab]
OR ECC[tiab] OR "tooth numbering"[tiab] OR mesiodens[tiab]
OR "dental age estimation"[tiab] OR MIH[tiab]
OR "molar incisor hypomineralization"[tiab])))

Filters applied:
- Publication date: 2015–2025
- Language: English
- Species: Humans

2. Scopus Search Strategy

(TITLE-ABS-KEY("artificial intelligence" OR "machine learning"
OR "deep learning" OR "neural network*" OR AI)
AND
TITLE-ABS-KEY("pediatric dentistry" OR "paediatric dentistry"
OR child* OR pediatric* OR paediatric*)
AND
TITLE-ABS-KEY("dental caries" OR caries OR "early childhood caries" OR ECC
OR mesiodens OR "tooth numbering" OR "age estimation"
OR MIH OR "molar incisor hypomineralization"))

Limits:
- Publication years: 2015–2025
- Document types: Articles, Reviews
- Language: English

3. Web of Science (Core Collection) Search Strategy

TS = (("artificial intelligence" OR "machine learning"
OR "deep learning" OR "neural network*" OR AI)
AND
("pediatric dentistry" OR "paediatric dentistry"
OR child* OR pediatric*)

AND
("dental caries" OR "caries detection"
OR "early childhood caries" OR ECC
OR mesiodens OR "supernumerary teeth"
OR "tooth numbering" OR "dental age estimation"
OR MIH OR "molar incisor hypomineralization"))

Indexes searched: SCI-Expanded, SSCI
Timespan: 2015–2025
Language: English

4. Embase Search Strategy

('artificial intelligence'/exp OR 'machine learning'/exp
OR 'deep learning'/exp OR 'neural network'/exp
OR 'artificial intelligence':ti,ab OR 'machine learning':ti,ab
OR 'deep learning':ti,ab OR 'neural network*':ti,ab)
AND
('pediatric dentistry'/exp OR child/exp
OR child*:ti,ab OR pediatric*:ti,ab OR paediatric*:ti,ab
OR 'pediatric dentistry':ti,ab OR 'paediatric dentistry':ti,ab)
AND
('dental caries'/exp OR 'early childhood caries'/exp
OR caries:ti,ab OR 'early childhood caries':ti,ab OR ECC:ti,ab
OR mesiodens:ti,ab OR 'supernumerary tooth'/exp
OR 'age estimation'/exp OR 'molar incisor hypomineralization'/exp
OR MIH:ti,ab)

Limits applied:
• Humans
• English
• 2015–2025

5. Additional Search Measures

- Reference lists of all included studies were screened.
- Reference lists of relevant systematic reviews were screened.
- Grey literature (Google Scholar) was searched for additional records.
